# The medical journal as an open access multimedia platform for medical communication

**DOI:** 10.3325/cmj.2020.61.483

**Published:** 2020-12

**Authors:** Domhnall MacAuley

**Affiliations:** Medical journal editorial consultant, Belfast, United Kingdom *domhnall.macauley@gmail.com*

What is the role of the medical journal in our current world? Traditionalists hold fast to the classic model where academics published strictly reviewed research and clinical data to a limited professional audience in a standard format creating a passive journal of record. But, communication has changed and medical journals will have to evolve or slowly become obsolete.

Expectations have changed. We now access information using electronic means almost exclusively. Most information is freely available and we become frustrated if we cannot access knowledge easily or if it is behind a pay wall. In medicine this is much more important than simply a matter of personal convenience. Public agencies and charities funding research expect outputs to be openly available and may mandate that research findings be published in open access journals. High profile journals are moving, although slowly, in this direction (1). There is also a gathering movement to make all research data open (2). It is difficult to argue against open access to publicly funded research where patients could benefit. Where patients have actively participated, it seems morally indefensible that research might not be openly accessible. In these circumstances, paywalls that benefit a medical society or independent publisher cannot be fair and just. The future is open access.

Research dissemination priorities have changed. In the past editors held all the power and researchers could only submit and hope that their work might be published. The route to research publication was narrow and controlled. The power balance is shifting, accelerated by the coronavirus disease 2019 (COVID-19) crisis, so that researchers are now more concerned that their research is available early and widely. New format journals offer a much wider publication flow using light touch peer review appraising only the method and without applying a value judgement. Preprints ensure research findings are available for public access before peer review and publication in a peer reviewed journal and, while this avoids delay through the peer review, it is not without faults (3). Interestingly, however, it also alters the researcher-editor relationship so that journals might soon find themselves competing to publish the best research in an open market. More recently, we have seen therapeutic and vaccine studies disseminated by press release without even a preprint, far in advance of peer reviewed publication (4). The future is in the hands of the researcher.

The audience has changed. Research published electronically is now accessed widely and immediately. Patients can access the latest research just as quickly as specialists in a field and the expert patient can help inform and determine their own personal treatment. This means that medical journals need to think about how they prioritise and package information for a much wider audience and many journals now involve patients in the publication decision process (5). The future will be consumer driven.

Information transfer has changed. We have become more passive consumers and now expect that information be packaged and delivered directly to us in more convenient formats. Research dissemination is increasingly important and scientists are much more aware of the different modalities (6). Researchers may even decide where to publish based on a journal’s dissemination strategy. As we become accustomed to accessing news and entertainment on multiple channels (including Twitter, Facebook, and Instagram), journals will have to respond and the future medical journal will be a multimedia medical communication platform disseminating research and education widely in text, audio, and video. With time, medical journals may find themselves pushed further down the information pathway so that, instead of being the primary provider of original research, they will curate, interpret, and prioritise research data released elsewhere, and package it for delivery to their consumers in different formats on multiple channels.

Medical journals are in the business of communication. Rapid changes in information dissemination mean that some journals, while conscientiously focused on improving the traditional journal model, have slipped out of step with modern communication practice. In keeping with the rest of the communication industry, medical journals will need to become more responsive, open, and accessible, focus on their changing audience, move from passive to active research dissemination, and create content in multiple formats. This is not about the future, it is about catching up with the present.
